# Gossypium Herbaceum (Cotton)

**Published:** 1861-04

**Authors:** 


### *Gossypium Herbaceum.*

### (COTTON)

If the Malvaceous plants, to which class the Gossypium belongs, are not especially remarkable for the energy of their medical properties, they are exceedingly interesting from the fact of the perfect uniformity that these properties present in the whole family. All Malvaceæ contain in their different parts a considerable quantity of mucilage; so they are essentially soothing and emollient, and can be employed indifferently, the one for the other, without the least inconvenience. In some countries they serve the purposes of food, as in many parts of Europe the young leaves of the mallows, when boiled, are eaten. In the two Indies and in Africa, the *Gombo*, or *Hibiscus esculentus*, is cultivated for the same purpose, the young fruit of which is esteemed highly nourishing.

There are, however, certain exceptions that are presented by this family. The leaves of the *Hibiscus sabduriferus* are acid, those of the *Sida lanceolata* and *mauritiana* are bitter and employed as febrifuges. The seeds of the *S. hirta* are narcotic, and those furnished by the *H. abelmoschus* are odorous, containing a colored resin and a volatile principle having an ordor like musk.

The Malvaceæ furnish a very important vegetable, the cotton plant, *Gossypium Herbaceum.* This species, and many others of the genus, have their fruit in capsules containing numerous seeds, whose integument is filled with long white or reddish filaments, soft, silky, known under the name of cotton. It is one of the great staple products of the country, as well as being one of the most important products of the commerce of the Indies with Europe. It is largely cultivated in North and South America, India, Africa, and the Antilles.

It has been disputed whether the varieties of the Gossypium are not all one species, and if these varieties are not owing to cultivation and the soil and climate of the countries where they are found. The extremes have been taken on the one side that they are all one, and on the other that they are many; however, the division generally adopted is triple, viz: *G. Herbaceum*, *G. Arboreum* and *G. Hirsutum*.

One peculiarity in the natural history of the cotton plant is that it does not arrive at great perfection in but few of the localities where it is indigenous. Some have gone so far as to say that it can be raised profitably and advantageously only in this country. In the wilds of Mexico, beyond the Del Norte, cotton is indigenous, or is supposed to be, and yet it cannot be *cultivated* there to advantage. It is known to grow in parts of that country, hundreds of miles from any habitation, and yet it requires to be transplanted to the Gulf of Mexico in order to be successfully cultivated.

The Herbaceous Cotton is stated to be of Eastern origin. It figures to a considerable extent in Classical and Egyptian history and probably is also referred to in Scriptural. It grows to the height of from eighteen to twenty-four inches, bearing leaves of dark green, blue veined and five lobed.—The flower is pale yellow, having one pistil, five petals, purple and spotted at the bottom. While this description of the color is true to an individual flower, still a field in full blossom presents every shade, from white to dark brown, passing through the various shades of yellow and red, not unfrequently varigated. On the falling of the flower a pod of triangular shape is developed. The pod, in the course of ripening, bursts, discloses a snow-white or yellowish ball of down in three locks, enclosing and tightly adhering to the seeds, which resemble those of the grape, though of several times the size. This species is the most useful, and said to be cultivated in nearly every country congenial to the Gossypium, existing at Aleppo, in Upper Egypt, Arabia and

Senegal. It is of indigenous American growth, for the Spaniards, on their first landing in Mexico, found it in considerable perfection, and sent a quantity of it to Spain, where it was manufactured into garments for the use of the grandees of the Court. The root is fusiform in shape, giving off small radicles throughout its length. The size of the root varies, according to the soil from which it is produced. Its length varies from a few inches to that of a foot. When the root is cut or broken, it displays a white color; the bark is of a reddish brown; the taste is pleasant, somewhat sweet and astringent; it contains more of the latter quality than the root from which it is procured; it is very mucilaginous in its properties. The root is easily broken when dry, but the bark is quite tenacious, pulling off in strings.

*Medical Properties.*—The cotton lint has been in quite extensive use in the treatment of blisters, scalds, burns, severe bruises and in rheumatic pains, being adopted by the profession from popular practice; but, with the exception of notices of the active properties of the root and seed, by Dr. Bouchelle, of Mississippi, in 1840; by Dr. Davis, of Monticello, S. C., in 1850, and by Dr. Travis., of Tennessee, in 1852, the plant has been well nigh unknown in the Materia Medica, and has been left almost wholly to the uses of Commerce. However, as is witnessed by latter experiments, the immense quantities of the root of the cotton plant, answering no useful purpose to the planter, but rather being regarded as an incumbrance to the free tillage of the soil, are possessed of great medicinal value. Dr. Bouchelle regarded it as an excellent emmenagogue, and not inferior to ergot in promoting uterine contraction. He stated that it was habitually and effectually resorted to by the slaves of the south for producing abortion, and this, too, without seriously affecting the general health. This emmenagogue property is its characteristic, acting with as much efficiency and more safety than ergot; operating without pain or gastric disturbance; producing no other effect than the excitation of the menstrual secretion, excepting, perhaps, some degree of anodyne influence.

Mr. Shaw, of Tennessee, writing to the *Nashville Journal*, says: "I consider this root one of the very best emmenagogues of the materia medica, and I think it should be so classed. My reasons for considering it such, are grounded upon the different experiments which I have made with it, within the last twelve months. I sometimes use a decoction, and at others an infusion, but most generally a decoction, prepared thus:

℞.—Cotton Root,........................4 ounces.
Water,..........................2 pounds.

Boil down to one pint. S.—A wine glass full every hour. This produces the most salutary effect in dysmenorrhœa; it acts as an anodyne in allaying the pain, and as an emmenagogue in aiding or augmenting menstruation; its action is very speedy; after its exhibition, in this case it produces an effect which, indeed, appears almost natural, that is, almost without pain; the patient, after its exhibition, feels but little inconveniences from pain, which soon subsides, and menstruation is immediately augmented, without acceleration of the pulse or gastric uneasiness. There are few other emmenagogues that can claim this feature.

Its action in amenorrhœa I think superior to any other emmenagogue belonging to the materia medica, though it would be proper to pay some attention to the general health of the patient before its exhibition. It is superior to anything that I have tried in the way of emmenagogues. I have had cases in which I first tried the usual emmenagogues, with but little effect, or success, when I would determine on trying the decoction of this root, which would far surpass my expectations by acting with the most marked effect; menstruation being produced on the following day after its exhibition. All of these symptoms disappeared on exhibition of this medicine. I believe this to be the best emmenagogue that we can employ in mere suppressio mensium, where there is no other disturbance in the general health.

With the usual emmenagogues, I was enabled to produce the catamenia on a young lady, which continued for about twenty-four hours, then suddenly becoming very sparce and painful; and in a few days after this period had passed, I employed the infusion of the cotton root as a means of exciting this function, which it did on the following day, a plentiful discharge being produced which continued for five or six days. She has been regular at every period since that time, and has enjoyed good health, with the exception of a few simple attacks which caused no derangement of the menstrual function. For about twelve months previous to the exhibition of this medicine, her health was very much impaired, but she commenced improving, and soon recovered health. I could detail other cases similar, in which I have tried the decoction with the same effect, but I deem it unnecessary to mention its action in each individual case.

*As a parturient agent*, I think it superior to ergot in one sense of the word, and in another about its equal, its action being about as prompt as that of ergot, and attended with much less danger. I have tried both in parturition, and found the cotton root decoction to act with fully as much efficacy as ergot. In some cases in which I have tried it, the pain was to some extent allayed, and labor promoted with as much speed as when ergot was administered. It appears to be perfectly harmless, from the fact that its action is almost unattended with pain. It causes neither gastric distress, or acceleration of the pulse; if it does, it is not perceptible; both of which are occasioned by ergot to some extent.

I have witnessed its action in retained placenta with good effect, which was an expulsion of the mass in about twenty minutes after the exhibition of the first dose. It may be proper to say, that I gave two doses before the placenta was thrown off. I believe it to be safer as a parturient agent, or an emmenagogue, or at least as safe, as any other article of the materia medica.

It should have a fair and impartial trial by the profession generally, because it will prove itself worthy of the time and labor spent in its investigation. It is handy to all, and free of expense. A few trials by the profession will confirm the truth of this short essay. Give it a trial, and it will prove itself in some cases of amenorrhœa, dysmenorrhœa, or probably in some lingering case of labor, which may require the assistance of medicine, to produce contraction of the uterus for the expulsion of the child. I think it worthy of the attention of the profession, in the above cases.

*Tincture of Cotton Root as a Tonic.*—There is a condition of the system in which this tincture acts as a valuable restorative. These cases are of a leuco-phlegmatic temperament of both sexes, but it is to the female sex that I wish to draw the attention of the reader. Where there is general bad health, accompanied with tardy menstruation, I have used it with the happiest effect; in a few cases of emansio mensium, caused by anemia, where the patient was troubled with pains in the loins and giddiness of the head, with a derangement of the digestive organs, such as anorexia, accompanied with an uneasy, depressed feeling at the scrobiculus cordis, every month, which was promptly relieved by the tincture, but not with the effect of producing the menstrual flux, which was afterwards produced by the decoction, I find it necessary to continue the tincture from two to four weeks. The strength of the tincture that I have been in the habit of using, is prepared thus:—

Bark of the Root, (dry,)................8 ounces.
Diluted Alcohol,.......................2 pounds.

Digest fourteen days, filter and give it in ℨj. doses, three or four times a day. The tincture which I used was prepared by myself; and as I have seen no account of its use, I claim the first preparation of it, as well as the first experiment with it. My brother, Dr. H. J. Shaw, has since tried it with the same good effect; in fact, his experience coincides with mine thoughout."

The cotton seed has acquired considerable reputation as an antiperiodic in cases of intermittent fever, the use of which originated with a planter in South Carolina, who says:—"I have never failed to cure a patient, with a single dose of it, even where large doses of quinine have failed."

It is however, in the peculiar active properties of the *root*, that the profession will be chiefly interested. The, oftentimes, great danger in administering ergot, prevents its use, even when its specific effects seem to be called for. If these specific effects can be obtained by the use of the cotton root, and this, too, without the liability of injury to the general system, and these have been attributed to it, the profession would do well to give it a thorough and extensive trial.—*Journal of Materia Medica.*

